# Red/green-light emission in continuous dielectric phase transition materials: [Me_3_NVinyl]_2_[MnX_4_] (X = Cl, Br)†

**DOI:** 10.1039/d0ra08795e

**Published:** 2021-01-11

**Authors:** Yanyan Li, Liting Lin, Jie Yang, Kun Qian, Tao Jiang, Hong Li

**Affiliations:** College of Pharmacy, Jiangxi University of Traditional Chinese Medicine Nanchang 330004 P. R. China qk0876@hotmail.com; Jiangxi Hosptial of Integrated Traditional Chinese and Western Medicine Nanchang 330003 P. R. China jllhong@163.com

## Abstract

The luminescence of dielectric phase transition materials is one important property for technological applications, such as low-energy electron excitation. The combination of dielectric phase transitions and luminescence within organic–inorganic hybrids would lead to a new type of luminescent dielectric phase transition multifunctional material. Here, we report two novel A_2_BX_4_ organic–inorganic hybrid complexes [Me_3_NVinyl]_2_[MnCl_4_] 1 and [Me_3_NVinyl]_2_[MnBr_4_] 2, ([Me_3_NVinyl] = trimethylvinyl ammonium cation). The complexes 1 and 2 were found to undergo continuous reversible phase transitions as well as switch dielectric phase transitions. Strikingly, intensive red luminescence and green luminescence were obtained under UV excitation respectively to reveal potential application of the two complexes in multi-functional materials along with dielectric switches and so on.

## Abstract 2

The luminescence of dielectric phase transition materials is one important property for technological applications, such as low-energy electron excitation.

## Graphical abstract

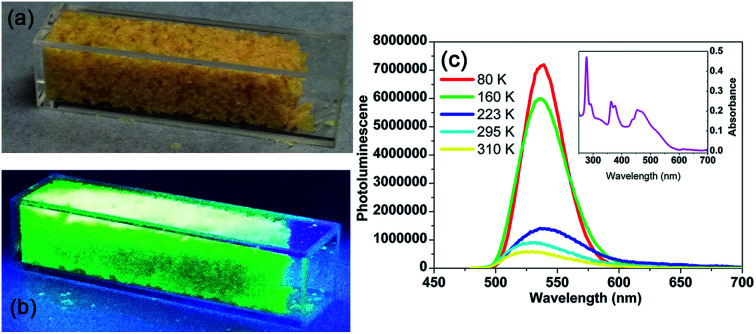

## 1. Introduction

Triggered by external stimuli like temperature, pressure and light, solid-state phase transitions are usually accompanied by dramatic variations in crystal structures and some physical properties, such as the thermal, optical, dielectric, magnetic and even ferroelectric properties.^1–9^ Consequently, phase transition materials have recently attracted a great deal of attention owing to their wide applications in data storage, signal processing, switchable dielectric devices and so on.^10–14^ Among these promising materials, dielectric materials with luminescence properties are unique, and can be applied to low-energy electron excitation. For example, the Al^3+^-added SrTiO_3_ : Pr^3+^ with a perovskite structure shows red cathodoluminescence, which can be excited at a low anode voltage; even below 10 V. The cathodoluminescence at such a low accelerating anode voltage makes this material attractive for potential applications in flat-panel display, field emission display (FED) and vacuum fluorescent displays.^15–17^

Along with the development and applications of organic–inorganic hybrid complexes in various fields in the past decade, a large number of luminescent materials based on hybrid organic–inorganic hybrid complexes have been developed because they combine facile synthesis with intriguing and tunable optical properties.^18^ These advantages make them suitable for a variety of low-cost optoelectronic devices, such as light emitting diodes^19–22^ and solar cells.^23–25^ Meanwhile, much progress has been achieved in ferroelectricity in organic–inorganic hybrid complexes.^26–40^ Our group reported a series of AMnX_3_-type luminescent ferroelectrics with excellent performances, including (pyrrolidinium)MnCl_3_, (pyrrolidinium)MnBr_3_, (3-pyrrolinium)MnCl_3_, and trimethylchloromethylammonium trichloromanganese(ii) (TMCM–MnCl_3_).^41–44^ Among them, TMCM–MnCl_3_ has shown a large piezoelectric coefficient *d*_33_ of up to 185 pC/N, which is comparable with that of classical piezoceramics, such as barium titanate (BTO; 190 pC/N).^44^ Their photoluminescence (PL) can be attributed to the ^4^T_1_–^6^A_1_ electronic transition of the octahedrally coordinated Mn^2+^ ions, which typically produces a red emission.^45^ Additionally, several luminescent molecular ferroelectrics based on lanthanide ions have been reported recently.^46–48^

In the search for new phase transition materials with excellent dielectric properties, quasi-spherical organic cations are usually selected as the building block for organic–inorganic hybrid complexes because they could trigger ordered-disordered phase transition and thus exhibit desired properties.^49–51^ The construction of molecular compounds with quasi-spherical organic cations has become a universal method for designing phase transition materials.^52^ Hybrid metal halide perovskite-type complex with general formula of A_2_BX_4_ was a good case (A = organic ammonium cation, B = divalent metal, X = halogen). Such hexagonal stacking perovskites involve a large class of complexes (M = Mn, Fe, Co, Ni, Cu, Cr, or V; X = Cl or Br).^53^ Among them, the Mn-complexes have been discovered to fluoresce brightly due to the existence of the luminous activator Mn^2+^ ion.^54^ Therefore, we tried to design A_2_BX_4_-type complexes, and found two new phase transition materials: [Me_3_NVinyl]_2_[MnX_4_] (X = Cl, Br), which exhibit intense luminescence respectively under an UV excitation. Here, their synthesis, structural phase transitions, and photoluminescence properties are described as follows.

## 2. Experimental

### 2.1 Synthesis

All reagents and solvents were commercially available without further purification. Trimethylvinyl ammonium chloride was synthesized according to the literature.^55^ Trimethylvinyl ammonium bromine was purchased from Sigma Aldrich with a purity of 99%. Complex 1 was obtained as block-shaped green crystals by slow evaporation of the mixture solution of manganese chloride and trimethylvinyl ammonium chloride in a molar ratio of 1 : 1 in methanol solution at room temperature, mp ∼ 230 °C. Complex 2 was synthesized as block-shaped orange single crystal by slow evaporation of the mixture solution of manganese bromide and trimethylvinyl ammonium bromide in a 40% hydrobromic acid solution, mp ∼ 250 °C. Single crystal suitable for X-ray diffraction analysis was selected and studied. Polycrystalline samples were prepared by grinding the air-dried crystals into fine powder. The exposure to air for complexes 1 and 2 is not good for testing due to hygroscopicity.

### 2.2 Single crystal structure determination

X-ray data for the title complex 1 was collected on a Bruker Smart APEX II diffractometer with Mo K_α_ radiation (*λ* = 0.71073 Å) by using an φ–ω scan mode at 200 K, 303 K and at 348 K. X-ray data for complex 2 was collected at 173 K and at 223 K. Data processing including empirical absorption corrections was performed using the Crystal Clear software package (Rigaku, 2005). Absorption corrections were applied using the Sadabs program.^56^ The structures were solved by direct methods^57^ with the Shelxtl program and refined by full matrix least-squares techniques on *F*^2^ with the Shelxtl program.^57–59^ Non-H atoms were refined anisotropically using all reflections with *I* > 2*σ*(*I*). All H atoms were generated geometrically and refined using a “riding” model with *U*_iso_ = 1.2 *U*_eq_ (C and N). The asymmetric units and the packing views were drawn with DIAMOND (Brandenburg and Putz, 2005). The angles and distances between some atoms were calculated using DIAMOND, and other calculations were carried out using SHELXLTL. For complex 1, a summary of the crystallographic data and detailed structure refinements at 200 K, 303 K and at 348 K are given in Table S1.† As for complex 2, crystallographic data and structure refinements at 173 K and at 223 K are listed in Table S5.† Selected bond lengths and bond angles were listed in Tables S2–S4† for complex 1. Selected bond lengths and bond angles were listed in Tables S6 and S7† for complex 2, respectively.

### 2.3 DSC measurements

Differential scanning calorimetry (DSC) experiments were performed on a Perkin-Elmer Diamond DSC instrument. The polycrystalline samples of complexes 1 and 2 were heated and cooled in aluminium crucibles with a rate of 20 K min^−1^ over the temperature ranges of 150–355 K and 160–240 K, respectively.

### 2.4 Dielectric measurements

The polycrystalline samples were pressed into pellets. The pressed-powder pellets deposited with silver conducting glue were used as electrodes in the dielectric measurements under an applied AC electric field of 1 V. For complex 1, the real parts (*ε*′) of the complex dielectric constants (*ε* = *ε*′ − i*ε*′′, where *ε*′′ represents the imaginary part) were measured on a Tonghui TH2828A impedance at various frequencies and within the temperature range of 180–280 K, 305–350 K, respectively. As for complex 2, the real parts (*ε*′) of the complex dielectric constants were measured at the respective frequencies of 1 kHz, 100 kHz and 1 MHz in the temperature range from 170 to 230 K.

### 2.5 Photoluminescence measurements

The ultraviolet spectra of powder samples for complexes 1 and 2 were obtained using UV-3600 plus spectrometer, within measurement range of 200 to 800 nm. And the fluorescence spectra for complexes 1 and 2 in solid states were measured on Shimadzu fluorescence spectrophotometer, recorded with excitation wavelength based on one of the strongest absorption band in the ultraviolet spectra. The quantum yield at room temperature was measured by Hamamatsu c9920-02G, Japan.

## 3. Results and discussion

### 3.1 Thermal properties

**Fig. 1:**
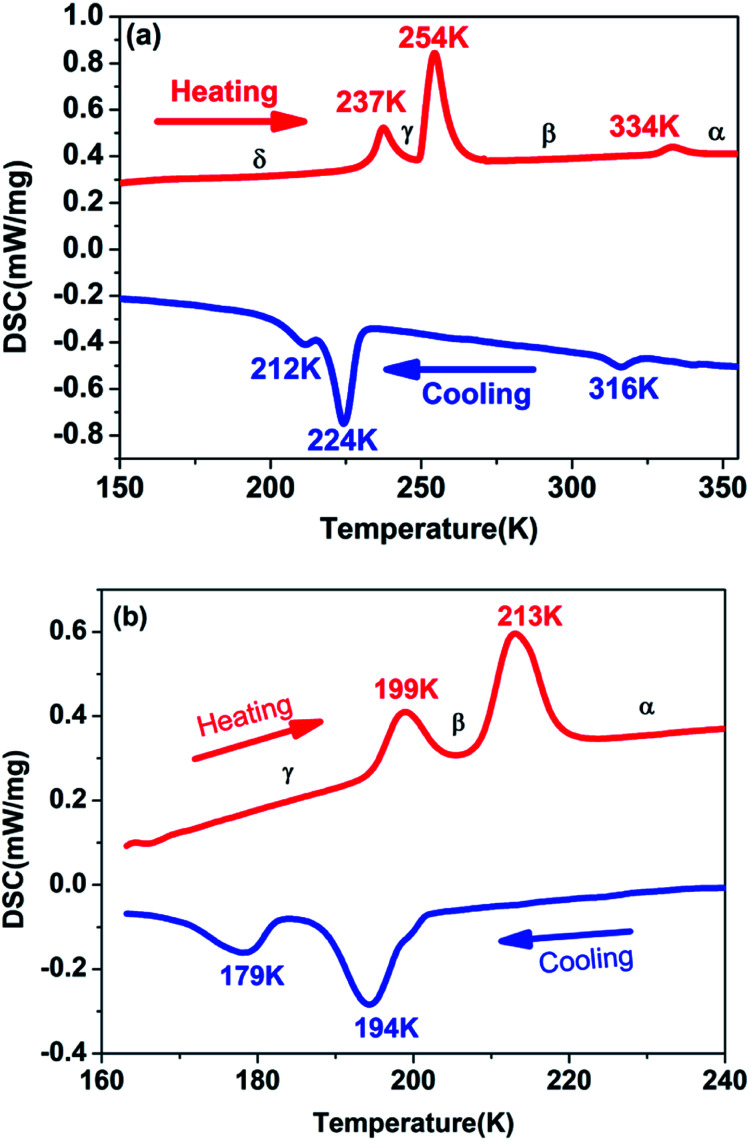
(a) DSC curves of complex 1 shown in the temperature range from 150 to 355 K. (b) DSC curves of complex 2 shown in the temperature range from 160 to 240 K.

Generally, DSC measurement is an effective and useful way to detect whether a crystal displays a reversible phase transition triggered by the external temperature. For the purpose of examining the phase transition characteristics of complexes 1 and 2, DSC measurements were carried out on them. As presented in Fig. 1a and b, DSC curves of complexes 1 and 2 were obtained for the heating and cooling cycles respectively. Despite the similar chemical formulas of [Me_3_NVinyl]_2_[MnCl_4_] (1) and [Me_3_NVinyl]_2_[MnBr_4_] (2), they displayed distinct phase transition temperatures (*T*_c_). For [Me_3_NVinyl]_2_[MnCl_4_] (1), complex 1 shows a small endothermic peak in the heating run at 334 K and a small exothermic peak in the cooling run at 316 K, indicating a reversible structural phase transition. Meanwhile, a couple of continuous reversible phase transitions below room temperature 254/224 K and 237/212 K were also observed in the DSC curve. For convenience, we take the transition temperature in the heating run as different phases, the temperature above 334 K as α phase, the temperature ranging from 254 to 334 K as β phase, the temperature ranging from 237 to 254 K as γ phase and the temperature below 237 K as δ phase.

For the complex 2, interestingly, and different from complex 1, [Me_3_NVinyl]_2_[MnBr_4_] (2) was found to only undergo two continuous reversible phase transitions below room temperature. A couple of continuous reversible phase transitions around the temperature 213/194 K and 199/179 K were observed in the DSC curve (Fig. 1b). For above room temperature, DSC measurements were carried out on them again with a rate of 3 K min^−1^. No phase transition was discovered above room temperature (Fig. S3†). For convenience, the phase above the temperature 213 K is designated as α phase, the phase between the temperature 199 and 213 K as β phase, and the phase below the temperature 199 K as γ phase.

### 3.2 Crystal structures discussion

**Fig. 2:**
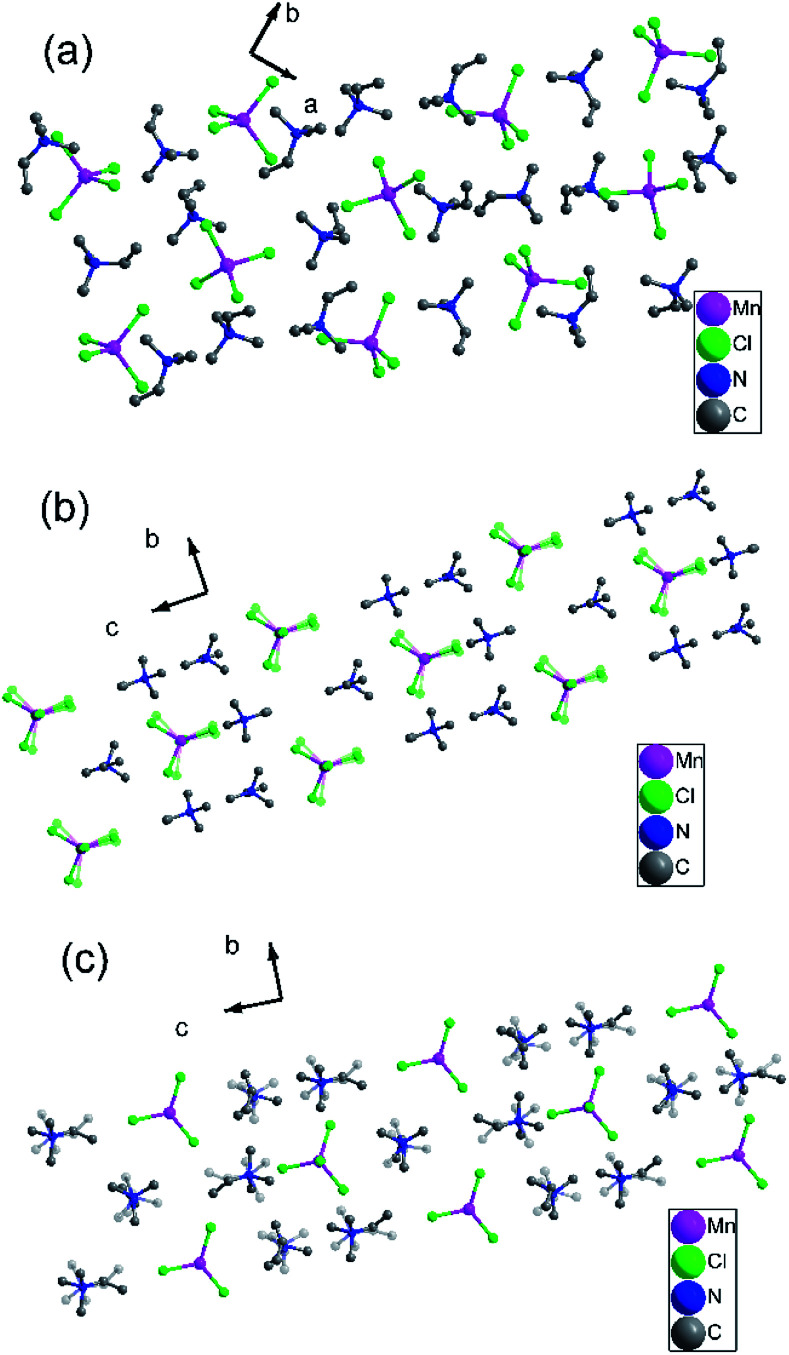
(a) Pack diagrams of complex 1 (200 K). (b) Pack diagrams of complex 1 (303 K). (c) Pack diagrams of complex 1 (348 K). Hydrogen atoms bonded to the C atoms were omitted for clarity.

To gain further insight into the details of the structural phase transitions of two complexes, variable-temperature X-ray structures of complex 1 were performed at 200, 303, and 348 K, and crystal structures of complex 2 were measured at 173 and 223 K, respectively. At 200 K (corresponding to δ phase), complex 1 crystallizes monoclinic crystal system *P*2_1_/*c* (no. 14), with cell parameters of *a* = 18.23(10) Å, *b* = 12.60 (4) Å, *c* = 18.13 (10) Å, *β* = 119.22(7)°, and *V* = 3637.2(4) Å^3^ (Table S1†). Each asymmetric unit consists of two [MnCl_4_]^2−^ anion and four trimethylvinyl ammonium cations, forming an organic–inorganic hybrid structure with a formula of [A_2_BX_4_]_2_ (Fig. S4a†). Each Mn atom is tetrahedrally coordinated by four bridged Cl atoms with Mn–Cl bond distances at the range from 2.344(19) to 2.375(2) Å and Cl–Mn–Cl angles of adjacent Cl atoms vary from 107.54(7) to 112.97(9)°. Moreover, the C=C bond distances are essentially identical, which are 1.281(9), 1.294(8), 1.299(9) and 1.304(7) respectively as shown in Table S2.† In the pack structure along plane *ab*, each Mn atom situates at 2^1^ axis, and the four symmetric operations (1, 2, *i*, *c*) in the δ phase could be observed, as shown in Fig. 2a.

When the temperature rises to 303 K (β phase), the structure parameters are remarkably dissimilar to those found in the δ-phase. At 303 K, complex 1 crystallizes in the orthorhombic system space group *Pnma* (no. 62). It is noteworthy that the molecular component in the β-phase is the same as that at 200 K in the δ-phase, while the basic unit of the crystal structure at 303 K has halved the content compared to that of the δ-phase at 200 K, with cell parameters of *a* = 13.13(9) Å, *b* = 8.89(10) Å, *c* = 16.20(14) Å, *β* = 90°, *Z* = 8 and the volume reducing twice from 3637.2(4) Å^3^ to 1889.5(3) Å^3^ (Table S1†). Each asymmetric unit consists of one [MnCl_4_]^2−^ anion and two trimethylvinyl ammonium cations, with a formula of [A_2_BX_4_] (Fig. S4b†). The rising in temperature gives rise to the change of *β* angle from 119.22(7)° to 90°, accompanied by the transition of crystal system from monoclinic to orthorhombic. In the β phase, the thermal ellipsoids of most atoms are accordingly larger than those in the δ phase, corresponding to a definitely disordered phase. With the temperature increasing from 200 K to 303 K, the swaying motions of the three chloride atoms are activated, while the ordered chloride atoms become disordered and occupy two positions, respectively (Fig. S4b†). The Mn–Cl bond distances at the range are from 2.303(10) to 2.342(4) Å and Cl–Mn–Cl angles of adjacent Cl atoms vary from 107.6(3) to 115.0(15)°. In the pack structure, all atoms are situated in a mirror plane along *a* axis as illustrated in Fig. 2b. During the transition from δ phase to β phase, the symmetry elements of the crystallographic symmetric operations (1, 2, *i*, *c*) have doubled to eight symmetric operations (1, 2, 2, 2, *i*, *a*, *m*, *n*) (Fig. S4b†).

Interestingly, with the rise of temperature, the structure at 348 K (α phase) still adopts the same orthorhombic system and the same space group *Pnma* with cell parameters of *a* = 13.10(14) Å, *b* = 9.04(12) Å, *c* = 16.18(19) Å, *β* = 90° and the volume expanding from 1889.5(3) Å^3^ to 1916.4(4) Å^3^. The asymmetric unit remains the same as that at 303 K in the β phase, consisting of one [MnCl_4_]^2−^ anion and two organic ammonium cations, with the same formula of A_2_BX_4_, as shown in Fig. S4c.† With the temperature increasing from 303 K to 348 K, the swaying motions of two trimethylvinyl ammonium cations are activated, while the ordered ammonium cations become disordered and occupy two positions, respectively (Fig. S4c†). The Mn–Cl bond distances are in the range of 2.325(3) to 2.353(4) Å and the angles Cl–Mn–Cl of adjacent Cl atoms vary from 107.94(10) to 111.62(16)°, as shown in Table S4.† Moreover, the C=C bond distances are larger than that in the β phase and δ phase, which are 1.342(9) and 1.337(9) Å. Compared with the β phase, the Mn–Cl bond distances and the Cl–Mn–Cl bond angles change slightly, resulting in the structure phase change, also confirmed by the DSC results. Although space group does not change from β phase to α phase, the structural phase transition of complex 1 belongs to isomorphic phase transition.

With the temperature rising from δ phase to α phase, the hydrogen bond networks of complex 1 changed, as shown in Fig. S5–S7.† At δ phase, each Cl atom was connected with hydrogen atom through H⋯Cl non-covalent interactions, forming rich hydrogen networks (Fig. S5†). When the temperature rising to 303 K (β phase), the H⋯Cl hydrogen bond shift between the order-disorder Cl atom. And only three Cl atoms are connected with hydrogen atoms through H⋯Cl hydrogen bond due to the vibration of Cl atom when the temperature rising (Fig. S6†). With the temperature rising to 348 K (α phase), the H⋯Cl hydrogen bond begins to shift among the trimethylvinyl ammonium cations (Fig. S7†).

**Fig. 3:**
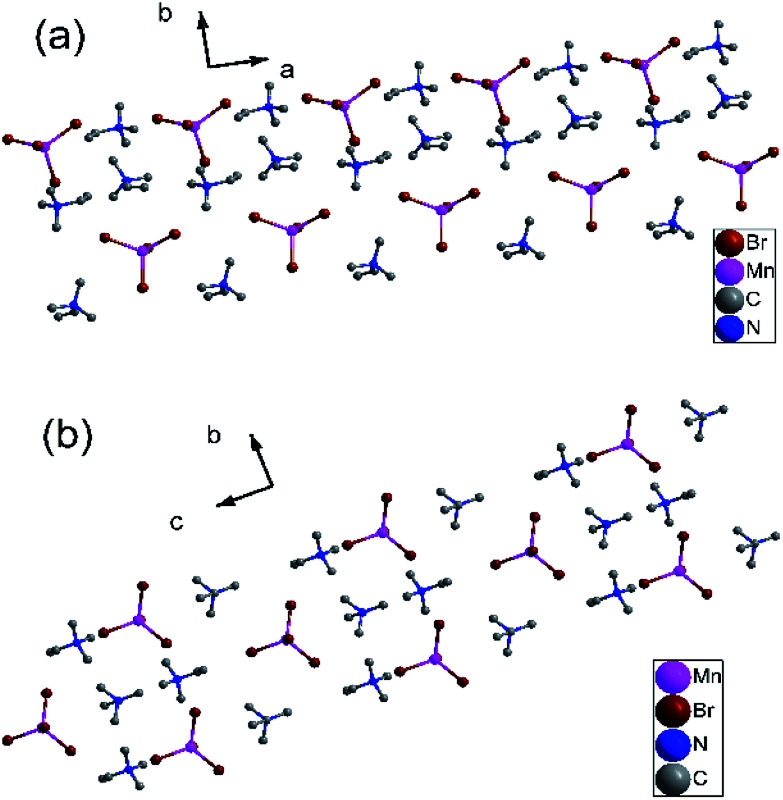
(a) Pack diagrams of complex 2 in the γ phase at 173 K. (b) Packing structure of complex 2 in the α phase at 223 K. All H atoms were omitted for clarity.

Similarly, the variable-temperature X-ray structures of complex 2 were performed at 173 and 223 K. At 173 K (γ phase), complex 2 crystallizes in the monoclinic crystal system the space group *P*2_1_/*c* (no. 14), with cell parameters of *a* = 9.23(6) Å, *b* = 15.19(10) Å, *c* = 13.74 (8) Å, *β* = 91.49(6)°, and *V* = 1926.2(2) Å^3^ (Table S5†). As shown in Fig. S8a,† each asymmetric unit is comprised of one [MnBr_4_]^2−^ anion and two trimethylvinyl ammonium cations. Each Mn atom is coordinated by four bridged Br atoms, forming a slightly distorted tetrahedral. The Mn–Br bond distances are at the range from 2.500(3) to 2.535(3) Å and Br–Mn–Br angles of adjacent Br atoms vary from 105.50(11) to 118.26(12)° (Table S6†). The four symmetric operations (1, 2, *i*, *c*) can be observed in the pack structure, as shown in Fig. 3a.

When the temperature rises to 223 K (α phase), complex 2 crystallizes in the orthorhombic system space group *Pnma* (no. 62), with cell parameters of *a* = 13.45(3) Å, *b* = 9.114(2) Å, *c* = 16.42(5) Å, *β* = 90°, and *V* = 2012.4(9) Å^3^ (Table S5†). Similarly, each asymmetric unit is comprised of one [MnBr_4_]^2−^ anion and two trimethylvinyl ammonium cations (Fig. S8b†). Each Mn atom is coordinated by four bridged Br atoms, forming a slightly distorted tetrahedral. Dissimilarly, all atoms are situated in a mirror plane along *a* axis in the pack diagram. (Fig. 3b) And the Mn–Br bond distances are at the range from 2.472(2) to 2.502(4) Å and Br–Mn–Br angles of adjacent Br atoms vary from 107.50(10) to 113.60(15)° (Table S7†). And the C=C bond distances decrease from 1.29(3) and 1.26(3) Å in the γ phase to 1.263(17) and 1.221(18) Å in the α phase. During the transition from γ phase to α phase, the symmetry elements of the crystallographic symmetric operations (1, 2, *i*, *c*) have doubled to eight symmetric operations (1, 2, 2, 2, *i*, *a*, *m*, *n*). Rich hydrogen bond networks of complex 2 are also observed in Fig. S9 and S10.†

### 3.3 Dielectric properties

Usually, the dielectric properties of crystalline materials exhibit distinctive anomalous changes of temperature-dependent dielectric constants in the vicinity of *T*_c_, while the magnitude of the variations is related to the characteristics of the phase transition. In consideration of the above-mentioned reversible phase transitions confirmed by the DSC and single crystal X-ray diffraction results, complexes 1 and 2 are expected to display interesting dielectric responses triggered by temperature.

**Fig. 4:**
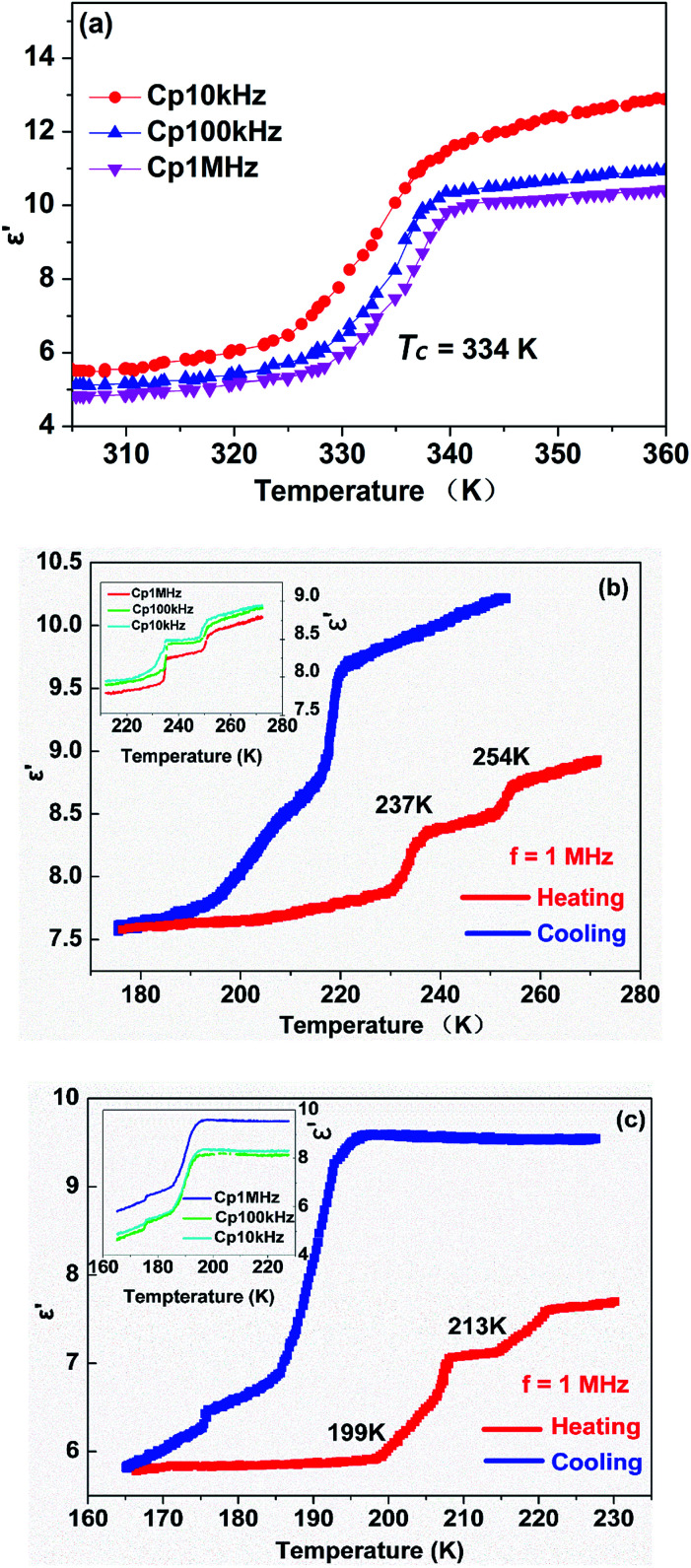
The dielectric responses of the polycrystalline samples of complexes 1 and 2 at various frequencies with variation of the temperature. (a) Temperature-dependence of the real part (*ε*′) of complex 1 at 10 kHz, 100 kHz and 1 MHz from β phase to α phase upon heating. (b) The real part (*ε*′) of complex 1 at 1 MHz from δ phase to β phase upon heating and cooling. Inset: the dielectric permittivity (*ε*′) of complex 1 at 10 kHz, 100 kHz, and 1 MHz upon heating. (c) The real part (*ε*′) of complex 2 at 1 MHz upon a heating–cooling cycle. Inset: the dielectric constant (*ε*′) of complex 2 at 1 kHz, 100 kHz and 1 MHz upon cooling.

In order to reflect the phase transitions more evidently, the dielectric properties of complex 1 were measured in the temperature range 180–280 K, 305–350 K at different frequencies, respectively (Fig. 4a and b). As illustrated in Fig. 4a, during the heating process at 1 MHz, the real part (*ε*′) has a value of about 5.9 when the temperature is ranging from 305 to 330 K, which is corresponded to β phase. At 334 K, the dielectric constant value (*ε*′) sharply increases. When the temperature is ranging from 340 to 350 K, the dielectric constant value (*ε*′) has a value of about 9.8, which is corresponded to α phase. That is, the magnitude of *ε*′ after phase transition is almost 1.6 times that before phase transition, demonstrating a noticeable step-like anomaly at around 334 K. Furthermore, the changes of *ε*′ at lower frequencies are more pronounced than those at higher frequencies, revealing that the dielectric constant is very sensitive to the external frequencies (Fig. 4a). The dielectric losses of complex 1 demonstrate a noticeable step-like anomaly at 334 K at various frequencies are also observed in Fig. S11.†

In case of the phase below room temperature, two pair of remarkable dielectric anomalies can be observed near the phase transition temperature. As displayed in Fig. 4b, upon heating the *ε*′ at 1 MHz remains about 7.6–7.9 from 180 to 231 K which is corresponded to δ phase, and then it increases to 8.4 at 237 K, seen as an apparent step-like anomaly around 237 K. The change of dielectric constant means the structural phase transition from δ phase to γ phase. With the temperature further increasing, the *ε*′ value increases slightly from 8.4 to 8.7 near 254 K, and the change of dielectric constant means the structural phase transition from γ phase to β phase. Furthermore, at 10 kHz and 100 kHz upon heating, the dielectric constant is sensitive to the external frequencies, consistent with the results recorded in Fig. 4b inset. Comparatively speaking, it is found that the dielectric response in the high temperature part is more sensitive to the change in frequency.

As for complex 2, the real part (*ε*′) at different frequencies upon a heating–cooling cycle shown in Fig. 4c further confirms the phase transition behaviours. Upon cooling, the *ε*′ kept stable with a value of about 9.6 when the temperature was decreasing from 227 to 196 K at 1 MHz, which corresponds to a high dielectric state. When further cooling, the *ε*′ value rapidly decreases to reach a value of about 6.9 at 186 K, transferring to a medium dielectric state with a step-like anomaly around the 194 K. Correspondingly, the change of dielectric constant means the structural phase transition from β phase to α phase in the heating process. The *ε*′ value at the high dielectric state is approximately 1.4 times as high as that at the medium dielectric state. Immediately, as temperature further declines, it displayed an abrupt decrease down to about 6.3 at 176 K, which corresponds to a low dielectric state. The change of dielectric constant equally means the structural phase transition from γ phase to β phase when heating. Furthermore, at 1 kHz, 100 kHz and 1 MHz upon cooling, the dielectric constant is sensitive to the external frequencies, consistent with the results recorded in Fig. 4c inset.

Overall, for complexes 1 and 2, the dielectric anomaly curves of the temperature-dependent *ε*′ obtained in the cooling modes match well with those recorded during the heating processes, strongly supporting the existence of reversible phase transitions, in good agreement with the DSC results mentioned above. Moreover, it is worth mentioning that the prominent step-like dielectric anomalies of the halogen substituted complexes also indicate the potential application prospects of dielectric switches in a tunable temperature range.

### 3.4 Photoluminescent properties

**Fig. 5:**
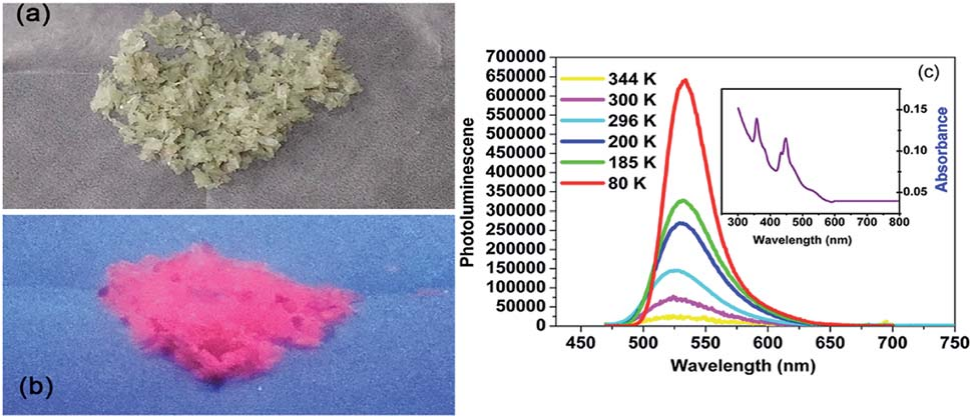
Photoluminescence properties of complex 1. (a) Image of the crystals of complex 1 under ambient light. (b) Image of the crystals of complex 1 under UV light. (c) Absorption (inset) and emission spectra of complex 1.

In addition to the dielectric properties, the optical absorption properties of complexes 1 and 2 were investigated by obtaining a solid state UV-vis transmission spectrum at room temperature. As shown in Fig. 5a, the crystals of complex 1 are pale green and somewhat transparent under ambient light. When subjected to UV lamp (365 nm), it shows intense red light as seen in Fig. 5b. In order to better characterize the luminescence property of complex 1, the absorption and emission spectra are as follows Fig. 5c. The absorption spectrum of the powder sample includes two strong absorption peaks (358 and 450 nm) in the ultraviolet region, which are related to the ^6^A_1_–^4^T_1_ electronic transition of Mn^2+^. Firstly, we measured the emission spectra at 296 K, and the emission spectra were recorded with excitation wavelength of 450 nm. The red phosphorescence at about 527 nm is produced by tetrahedrally coordinated manganese, which is attributable to the (e)^2^(t_2_)^3^–(e)^1^(t_2_)^4^ electronic transition.^60^

Then by quenching the heated components, the luminescence data were collected at 80, 185, 200, 300 and 344 K, respectively. As shown in Fig. 5c, the complex 1 exhibited the strongest emission at 80 K. However, the emission intensity decreased sharply with the temperature rising from 80 to 344 K, which might be ascribed to the non-radiation relaxation increased at high temperature. It should be noted that the luminescence peaks were also different with different symmetries. When the temperature was rose slowly from 80 to 185 and 200 K (δ phase), the emission peak was slightly shifted from 534 to 532 nm. Interesting, when the temperature rising to 300 K (β phase) and 344 K (α phase), the emission maximum was hypsochromic-shift by 10 nm to 524 nm. This may be ascribed to the changed intermolecular stacking mode, owning to the molecular of complex 1 was more and more disorder when the temperature increased from 80 to 300 K. By exciting the sample at 450 nm, the quantum yield at room temperature is 2%.

**Fig. 6:**
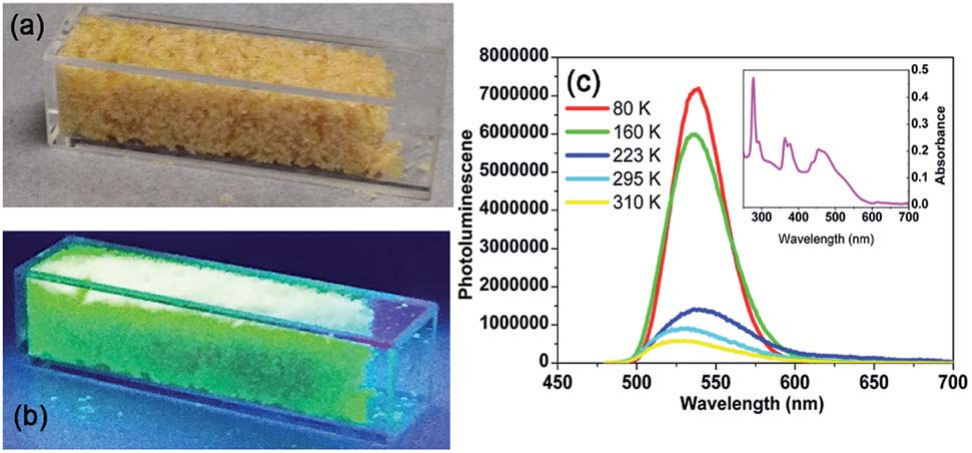
Photoluminescence properties of complex 2. (a) Image of the crystals of 2 under ambient light. (b) Image of the crystals of 2 under UV light. (c) Absorption (inset) and emission spectra of 2.

For complex 2, when subjected to a UV lamp at 365 nm, the orange crystals emit intense green light, as shown in Fig. 6a and b. The absorption spectrum consists of several bands ranging from visible to UV, as shown in the UV-vis result in Fig. 6c. By resonantly exciting the sample at 460 nm (on one of the strongest absorption band in visible range), the photoluminescence (PL) spectra at room temperature was exhibited in Fig. 6c. In the PL spectra, a strong emission can be observed at about 530 nm at 295 K. Such emission originates from the tetrahedrally coordinated manganese, where the detailed discussion can be found in Orgel's work in 1958.^60^

Then by quenching the heated components, the luminescence data of complex 2 were collected at 80, 160, 223 and 310 K, respectively. As shown in Fig. 6c, the complex 2 exhibited the strongest emission at 80 K. When the temperature was rising from 80 to 160 K (γ phase), the strongest fluorescence peak shifted from 539 to 537 nm. When the temperature was rising to 223 K (α phase), the strongest fluorescence peak shifted from 537 to 536 nm. Interesting, when the temperature was rising to 310 K (α phase), the emission maximum was shift to 530 nm again. With the temperature rising from 80 to 300 K, intermolecular stacking mode changed, lead to the emission maximum hypsochromic-shift. By exciting the sample at 460 nm, the quantum yield at room temperature is 4.2%.

**Fig. 7:**
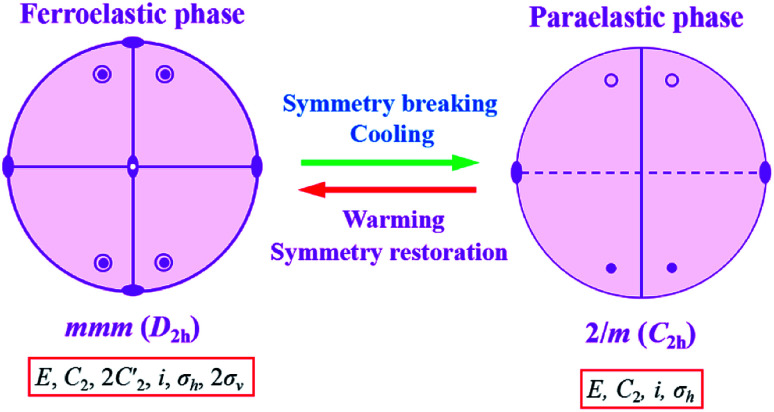
Symmetric breaking diagram of complexes 1 and 2.

In addition, we noticed that the point group changes from point group 2/*m* (*P*2_1_/*c*) to point group *mmm* (*Pnma*) belonging to the 94 species of ferroelastic phase transitions with an *Aizu* notation of *mmm F*2/*m* in complexes 1 and 2, corresponding to one ferroelastic–paraelastic phase transitions. The orthorhombic–monoclinic transition lowers the symmetry from the *mmm* to the 2/*m*. Eight symmetry elements in *mmm* decrease to four in 2/*m*. Hence the possible domain number is 8/4, that is, two. The Curie symmetry principle tells us that the space group at the ferroelastic phase should be the subspace group at the paraelastic phase. The number of spatial symmetry operations decreases from 8 to 4 during the symmetry breaking process, as shown in Fig. 7. The explore of ferroelastic domain will be carried out in next step.

## 4. Conclusions

In summary, the present work has successfully demonstrated two A_2_BX_4_ novel organic–inorganic hybrid complexes: [Me_3_NVinyl]_2_[MnX_4_] (X = Cl, Br), which exhibit multiple structural phase transitions along with photoluminescent properties. Complexes 1 and 2 underwent continuous reversible phase transitions, which were confirmed by the DSC results, variable-temperature structural analyses and dielectric measurements. The results indicate that the dielectric phase transitions are related to the motion of ions under the stimuli of variable temperature. It is worth mentioning that accompanying the phase transitions, complex 1 and 2 also display notable step-like dielectric anomalies, which are characteristics of switchable dielectrics. Additionally, the two complexes reveal striking intensive fluorescence properties with strong emissions observed in the vicinity of 540 nm. Combining the photoluminescent property, structural flexibility and dielectric properties, it is expected that our findings will not only throw light on the search for new dielectric photoluminescent materials but also accelerate their potential practical applications such as dielectric switch, *etc.*

## Conflicts of interest

There are no conflicts to declare.

## Supplementary Material

RA-011-D0RA08795E-s001

RA-011-D0RA08795E-s002

## References

[cit1] Fu D. W., Cai H. L., Liu Y. M., Ye Q., Zhang W., Zhang Y., Chen X. Y., Giovannetti G., Capone M., Li J. Y., Xiong R. G. (2013). Science.

[cit2] Shi P. P., Ye Q., Li Q., Wang H. T., Fu D. W., Zhang Y., Xiong R. G. (2014). Chem. Mater..

[cit3] Ye Q., Akutagawa T., Hoshino N., Kikuchi T., Noro S.-I., Xiong R. G., Nakamura T. (2011). Cryst. Growth Des..

[cit4] Ye Q., Akutagawa T., Endo T., Noro S.-I., Nakamura T., Xiong R. G. (2010). Inorg. Chem..

[cit5] Ye Q., Shi P. P., Chen Z. Q., Akutagawa T., Noro S.-I., Nakamura T. (2012). Eur. J. Inorg. Chem..

[cit6] Kong L. H., Fu D. W., Ye Q., Ye H. Y., Zhang Y., Xiong R. G. (2014). Chin. Chem. Lett..

[cit7] Hoshino N., Takeda T., Akutagawa T. (2014). RSC Adv..

[cit8] Akutagawa T., Koshinaka H., Sato D., Takeda S., Noro S.-I., Takahashi H., Kumai R., Tokura Y., Nakamura T. (2009). Nat. Mater..

[cit9] Shang R., Chen S., Wang Z. M., Gao S. (2014). Chem.–Eur. J..

[cit10] Vogelsberg C. S., Garcia-Garibay M. A. (2012). Chem. Soc. Rev..

[cit11] Coskun A., Banaszak M., Astumian R. D., Stoddart J. F., Grzybowski B. A. (2012). Chem. Soc. Rev..

[cit12] Lencer D., Salinga M., Wuttig M. (2011). Adv. Mater..

[cit13] Wuttig M., Yamada N. (2007). Nat. Mater..

[cit14] Zhang Y., Ye H. Y., Cai H. L., Fu D. W., Ye Q., Zhang W., Zhou Q. H., Wang J. L., Yuan G. L., Xiong R. G. (2014). Adv. Mater..

[cit15] Yamamoto H., Okamoto S., Kobayashi H. (2002). J. Lumin..

[cit16] Pizani P. S., Leite E. R., Pontes F. M., Paris E. C., Rangel J. H., Lee E. J. H., Longo E., Delega P., Varela J. A. (2000). Appl. Phys. Lett..

[cit17] Yamamoto H., Okamoto S. (2000). Displays.

[cit18] Xu H., Chen R., Sun Q., Lai W., Su Q., Huang W., Liu X. (2014). Chem. Soc. Rev..

[cit19] Tan Z. K., Moghaddam R. S., Lai M. L., Docampo P., Higler R., Deschler F., Price M., Sadhanala A., Pazos L. M., Credgington D., Hanusch F., Bein T., Snaith H. J., Friend R. H. (2014). Nat. Nanotechnol..

[cit20] Hang X. C., Fleetham T., Turner E., Brooks J., Li J. (2013). Angew. Chem., Int. Ed..

[cit21] Dohner E. R., Jaffe A., Bradshaw L. R., Karunadasa H. I. (2014). J. Am. Chem. Soc..

[cit22] Dohner E. R., Hoke E. T., Karunadasa H. I. (2014). J. Am. Chem. Soc..

[cit23] Chen C. Y., Chen J. G., Wu S., Li J. Y., Wu C. G., Ho K. C. (2008). Angew. Chem., Int. Ed..

[cit24] Pellet N., Gao P., Gregori G., Yang T. Y., Nazeeruddin M. K., Maier J., Gratzel M. (2014). Angew. Chem., Int. Ed..

[cit25] Xing G. C., Mathews N., Sun S. Y., Lim S. S., Lam Y. M., Gratzel M., Mhaisalkar S., Sum T. C. (2013). Science.

[cit26] Du Z. Y., Xu T. T., Huang B., Su Y. J., Xue W., He C. T., Zhang W. X., Chen X. M. (2015). Angew. Chem., Int. Ed..

[cit27] Xu G. C., Ma X. M., Zhang L., Wang Z. M., Gao S. (2010). J. Am. Chem. Soc..

[cit28] Xu G. C., Zhang W., Ma X. M., Chen Y. H., Zhang L., Cai H. L., Wang Z. M., Xiong R. G., Gao S. (2011). J. Am. Chem. Soc..

[cit29] Zhang W., Xiong R. G. (2012). Chem. Rev..

[cit30] Du Z. Y., Zhao Y. P., Zhang W. X., Zhou H. L., He C. T., Xue W., Wang B. Y., Chen X. M. (2014). Chem. Commun..

[cit31] Samantaray R., Clark R. J., Choi E. S., Dalal N. S. (2012). J. Am. Chem. Soc..

[cit32] Jain P., Ramachandran V., Clark R. J., Zhou H. D., Toby B. H., Dalal N. S., Kroto H. W., Cheetham A. K. (2009). J. Am. Chem. Soc..

[cit33] Di Sante D., Stroppa A., Jain P., Picozzi S. (2013). J. Am. Chem. Soc..

[cit34] Pan L., Liu G., Li H., Meng S., Han L., Shang J., Chen B., Platero-Prats A. E., Lu W., Zou X. D., Li R. W. (2014). J. Am. Chem. Soc..

[cit35] Liu C. M., Xiong R. G., Zhang D. Q., Zhu D. B. (2010). J. Am. Chem. Soc..

[cit36] Stroppa A., Jain P., Barone P., Marsman M., Perez-Mato J. M., Cheetham A. K., Kroto H. W., Picozzi S. (2011). Angew. Chem., Int. Ed..

[cit37] Stroppa A., Barone P., Jain P., Perez-Mato J. M., Picozzi S. (2013). Adv. Mater..

[cit38] Tian Y., Stroppa A., Chai Y. S., Yan L. Q., Wang S. G., Barone P., Picozzi S., Sun Y. (2014). Sci. Rep..

[cit39] Tian Y., Stroppa A., Chai Y. S., Barone P., Perez-Mato M., Picozzi S., Sun Y. (2015). Phys. Status Solidi RRL.

[cit40] Draxl C., Nabok D., Hannewald K. (2014). Acc. Chem. Res..

[cit41] Zhang Y., Liao W. Q., Fu D. W., Ye H. Y., Chen Z. N., Xiong R. G. (2015). J. Am. Chem. Soc..

[cit42] Zhang Y., Liao W. Q., Fu D. W., Ye H. Y., Liu C. M., Chen Z. N., Xiong R. G. (2015). Adv. Mater..

[cit43] Ye H. Y., Zhou Q. H., Niu X. H., Liao W. Q., Fu D. W., Zhang Y., You Y. M., Wang J. L., Chen Z. N., Xiong R. G. (2015). J. Am. Chem. Soc..

[cit44] You Y. M., Liao W. Q., Zhao D. W., Ye H. Y., Zhang Y., Zhou Q. H., Niu X. H., Wang J. L., Li P. F., Fu D. W., Wang Z. M., Gao S., Yang K. L., Liu J. M., Li J. Y., Yan Y. F., Xiong R. G. (2017). Science.

[cit45] Blasse G. and Grabmaier B. C., Luminescent Materials, Springer-Verlag, Berlin Heidelberg, 1st edn, 1994

[cit46] Guo P. H., Meng Y., Chen Y. C., Li Q. W., Wang B. Y., Leng J. D., Bao D. H., Jia J. H., Tong M. L. (2014). J. Mater. Chem. C.

[cit47] Guo P. H., Liu J. L., Jia J. H., Wang J., Guo F. S., Chen Y. C., Lin W. Q., Leng J. D., Bao D. H., Zhang X. D., Luo J. H., Tong M. L. (2013). Chem.–Eur. J..

[cit48] Long J., Rouquette J., Thibaud J. M., Ferreira R. A. S., Carlos L. D., Donnadieu B., Vieru V., Chibotaru L. F., Konczewicz L., Haines J., Guari Y., Larionova J. (2015). Angew. Chem., Int. Ed..

[cit49] Zhang H. Y., Tang Y. Y., Shi P. P., Xiong R. G. (2019). Acc. Chem. Res..

[cit50] Hajlaoui S., Chaabane I., Oueslati A., Guidara K. (2013). Solid State Sci..

[cit51] Ye H. Y., Li S. H., Zhang Y., Zhou L., Deng F., Xiong R. G. (2014). J. Am. Chem. Soc..

[cit52] Sun Z., Liu X., Khan T., Ji C., Asghar M. A., Zhao S., Li L., Hong M., Luo J. (2016). Angew. Chem., Int. Ed..

[cit53] Ackerman J. F., Cole G. M., Holt S. L. (1974). Inorg. Chim. Acta.

[cit54] Morosin B., Graeber E. J. (1967). Acta Crystallogr..

[cit55] Pernak J., Niemczak M., Zakrocka K., Praczyk T. (2013). Tetrahedron.

[cit56] Sheldrick G. M. , Sadabs, Program for Empirical Absorption Correction of Area Detector Data, University of Göttingen, Göttingen (Germany), 1996

[cit57] Sheldrick G. M. , Shelxtl (version 6.1), Software Reference Manual, Bruker AXS Inc, Madison, Wisconsin (USA), 2000

[cit58] Sheldrick G. M. (1990). Acta Crystallogr., Sect. A: Found. Crystallogr..

[cit59] Sheldrick G. M. (2008). Acta Crystallogr., Sect. A: Found. Crystallogr..

[cit60] Orgel L. E. (1955). J. Chem. Phys..

