# Supplementary material for: Red/green-light emission in continuous dielectric phase transition materials: [Me3NVinyl]2[MnX4] (X = Cl, Br)
Source: RSC Adv. 2021 Jan 11;11(4):2329–36. doi: 10.1039/d0ra08795e (PMC8693754; doi:10.1039/d0ra08795e)
Supplement: RA-011-D0RA08795E-s001 [file RA-011-D0RA08795E-s001.pdf]

Electronic Supplementary Material (ESI) for RSC Advances.
This journal is © The Royal Society of Chemistry 2020
Please do not adjust margins

Red/Green-light emission in continuous dielectric phase transitions materials: $[Me_3NVinyl]_2[MnX_4]$ (X = Cl, Br)

Yanyan Li,[a] Liting Lin,[a] Jie Yang,[a] and Kun Qian[a,*]

a. College of Pharmacy, Jiangxi University of Traditional Chinese Medicine, Nanchang, 330004, P. R. China, e-mail: qk0876@hotmail.com

Synthesis of the title complexes

Synthesis of the complex 1

Trimethylvinyl ammonium chloride (5 mmol, 0.612 g) and manganese chloride (5 mmol, 0.629 g) were dissolved in 12mL methanol solution with stirring. The resultant clear solution was allowed to stand at room temperature. Two days later, green block-shaped crystals of complex **1** were deposited from the solution in approximately 37.5% yield based on Mn. -Anal. calcd. (%) for $C_{10}H_{24}N_2Cl_4Mn$: C 32.54, H 6.55, N 7.59; found C 32.78, H 8.05, N 7.26. – FT-IR (KBr, cm^{-1}): 3450(w), 3110(m), 3026(s), 3000(m), 1660(s), 1462(s), 1412(m), 1248(w), 1070(w), 954(s), 896(s), 710(m).

Synthesis of the complex 2

Trimethylvinyl ammonium bromine (5 mmol, 0.830 g) and manganese bromide (5 mmol, 1.074 g) were dissolved in 20mL of 40% hydrobromic acid solution with stirring. The resultant clear solution was allowed to place at the temperature 48°C. One week later, orange block-shaped crystals of complex **2** were deposited from the solution in approximately 22.5% yield based on Mn. -Anal. calcd. (%) for $C_{10}H_{24}Br_4MnN_2$: C 21.96, H 4.42, N 5.12; found C 22.03, H 4.55, N 5.22. – FT-IR (KBr, cm^{-1}): 3462(w), 3112(m), 3024(s), 3004(m), 1660(s), 1486(s), 1412(m), 1246(w), 1066(w), 952(s), 894(s), 706(m).

Please do not adjust margins

Please do not adjust margins

Figure Captions

Fig. S1 Infrared spectrum of solid complex **1** in KBr pellets recorded at room temperature.

Fig. S2 Infrared spectrum of solid complex **2** in KBr pellets recorded at room temperature.

Fig. S3 DSC curves of complex **2** obtained in the heating-cooling cycles from 280 to 400 K.

Fig. S4 Molecular structure diagrams of complex **1** shown at different temperatures. (a) 200K, (b) 303 K, (c) 348 K

Fig. S5 Hydrogen network diagrams of complex **1** at 200 K

Fig. S6 Hydrogen network diagrams of complex **1** at 303 K

Fig. S7 Hydrogen network diagrams of complex **2** at 348 K

Fig. S8 Molecular structure diagrams of complex **2** shown at different temperatures. (a) 173K, (b) 223K

Fig. S9 Hydrogen network diagrams of complex **2** at 173 K

Fig. S10 Hydrogen network diagrams of complex **2** at 223 K

This journal is © The Royal Society of Chemistry 2020

Please do not adjust margins

Please do not adjust margins

Fig. S11 The dielectric losses of complex **1** at various frequencies with variation of the temperature.

Fig. S12 DSC curves of complex **1** obtained in three heating-cooling cycles.

Fig. S13 DSC curves of complex **2** obtained in three heating-cooling cycles.

This journal is © The Royal Society of Chemistry 2020

Please do not adjust margins

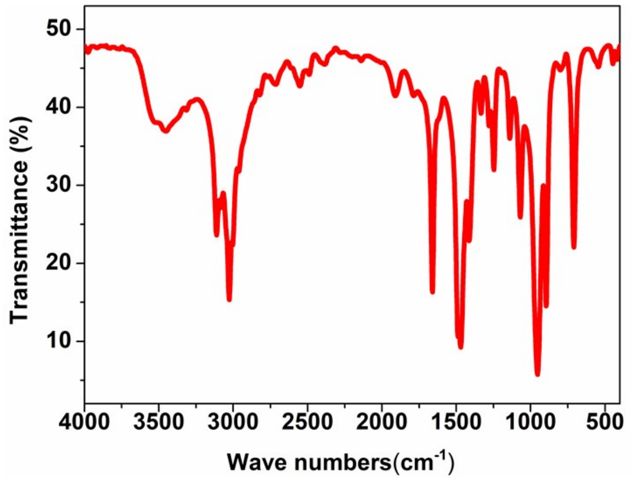

Figure S1

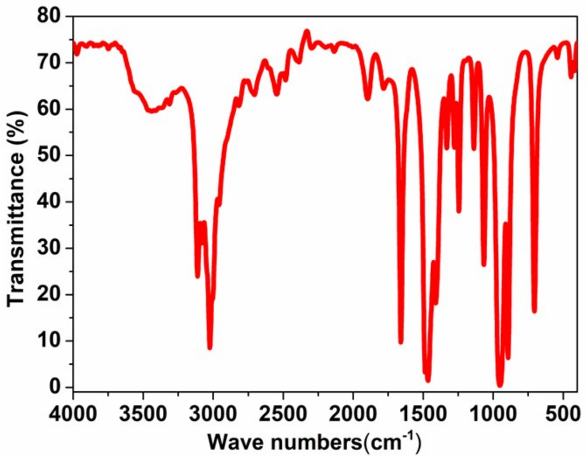

Figure S2

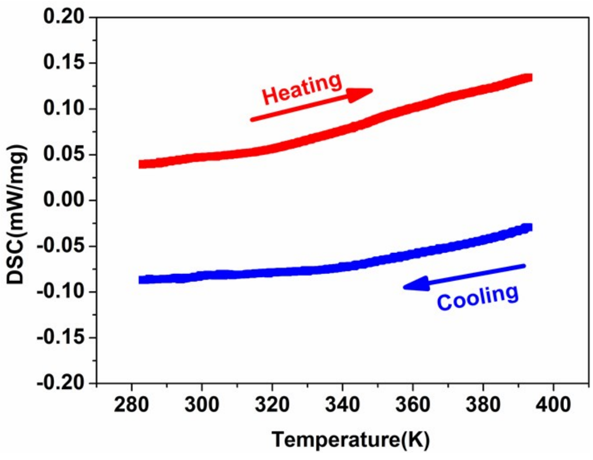

Figure S3

Please do not adjust margins

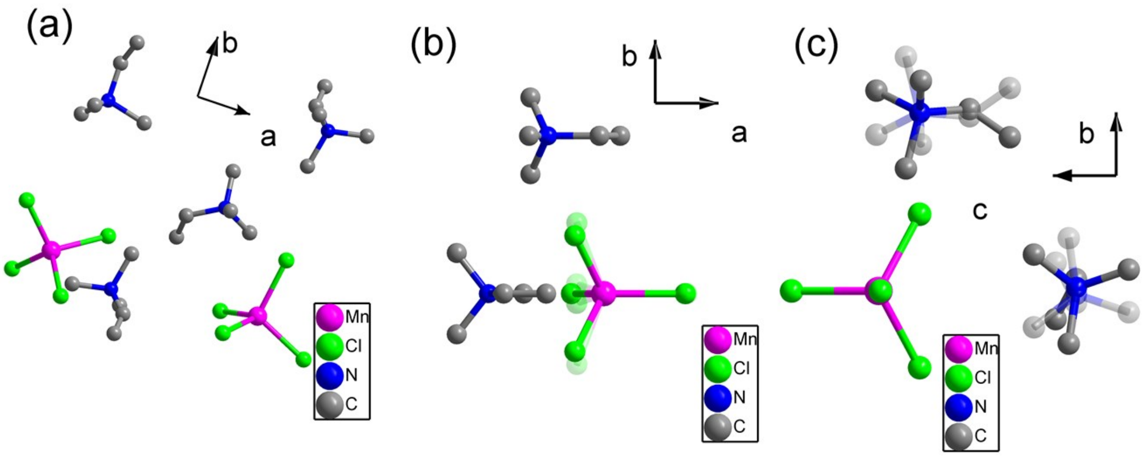

Figure S4

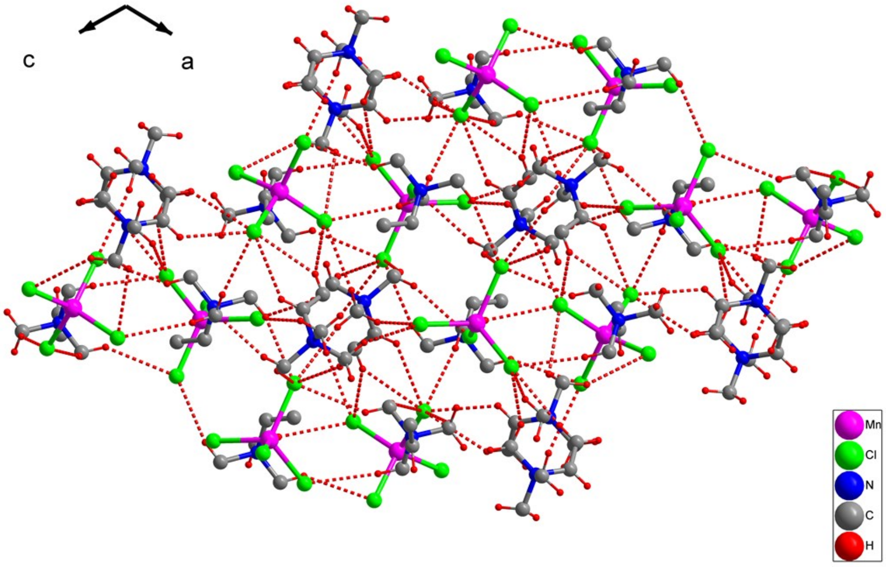

Figure S5

This journal is © The Royal Society of Chemistry 2020

Please do not adjust margins

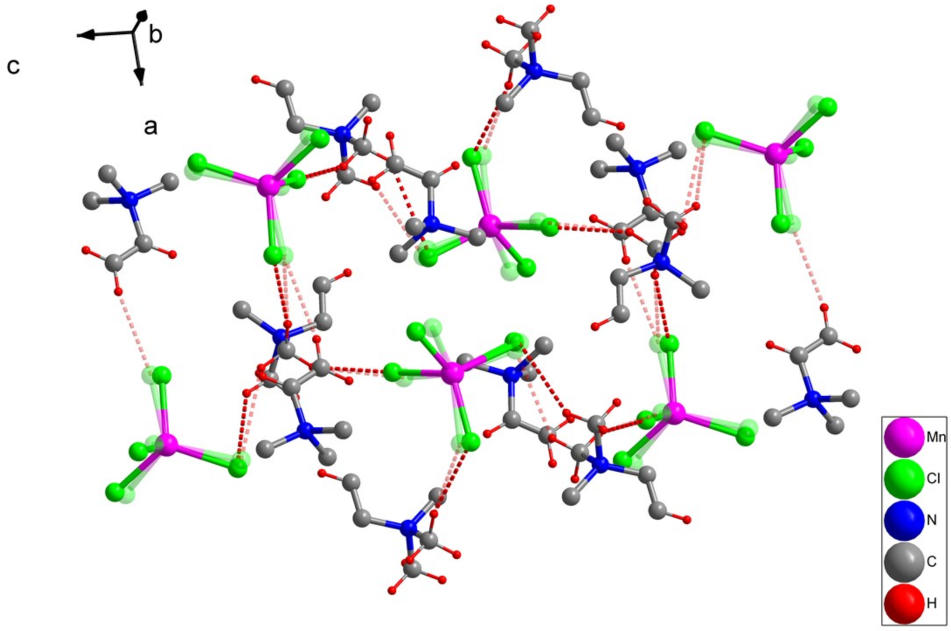

Figure S6

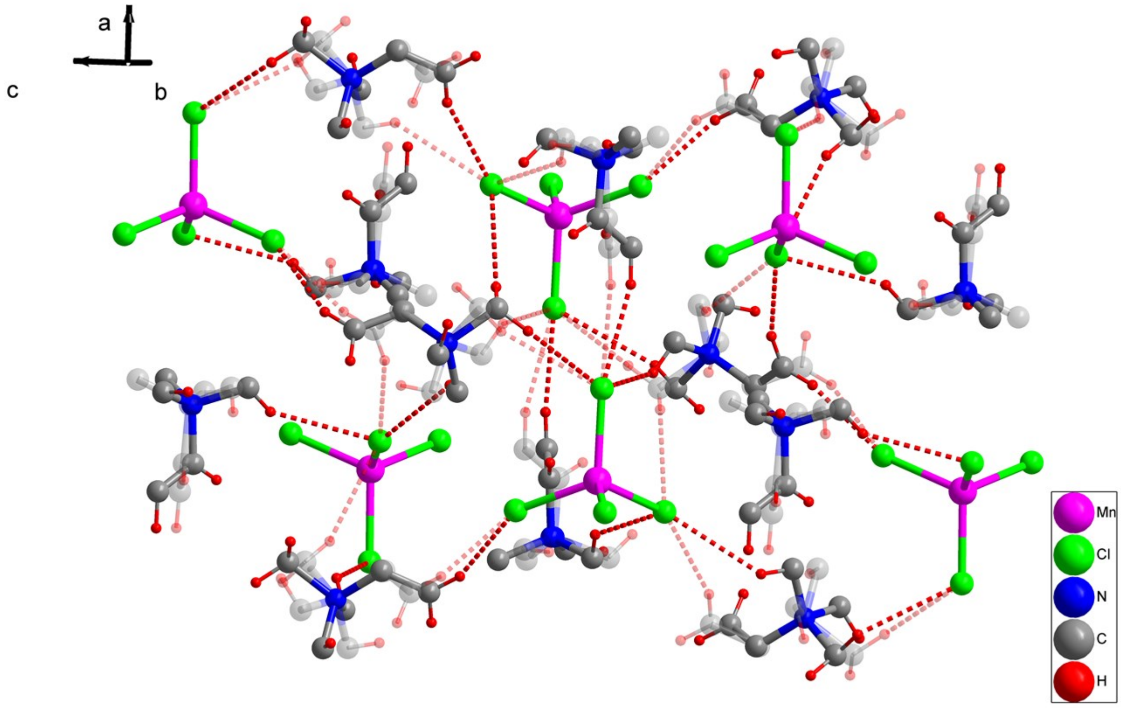

Figure S7

This journal is © The Royal Society of Chemistry 2020

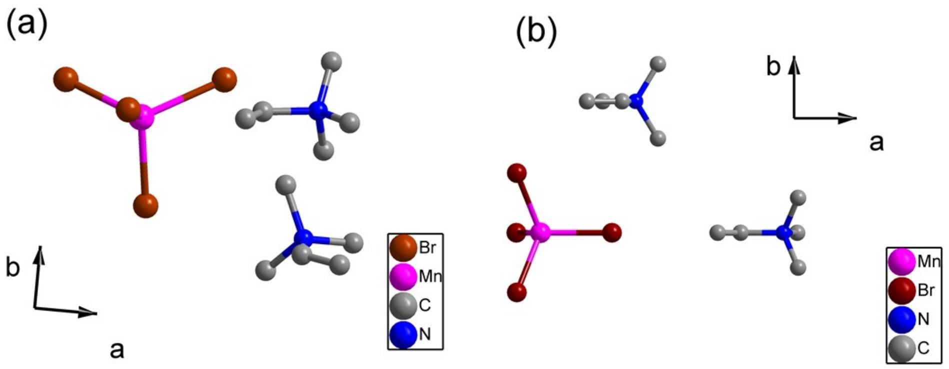

Figure S8

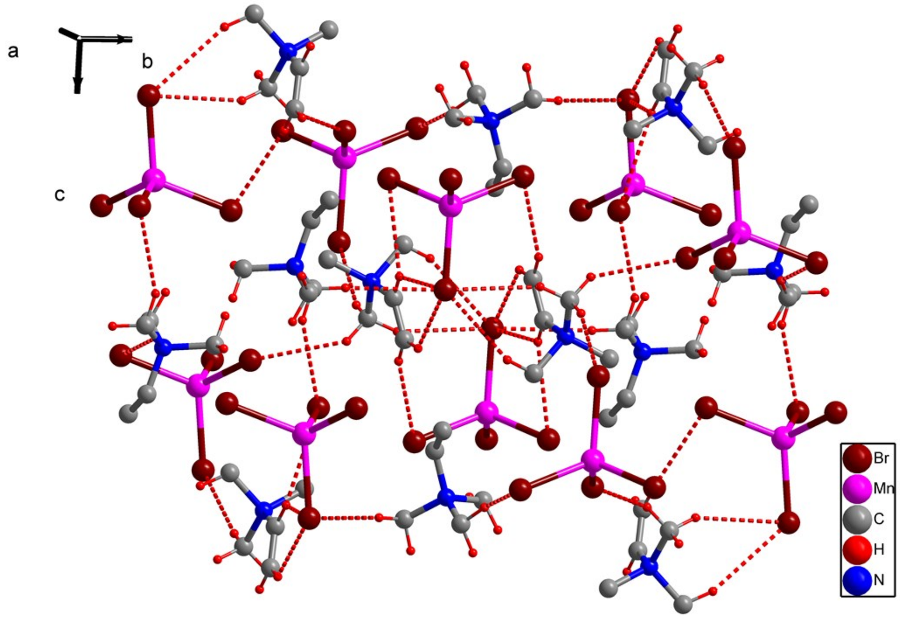

Figure S9

Please do not adjust margins

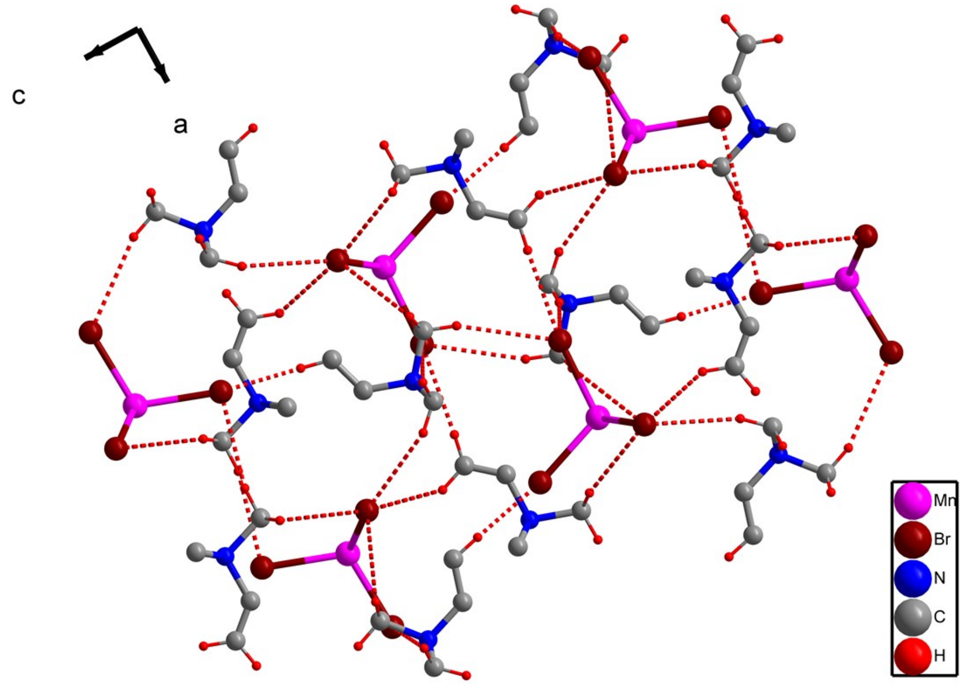

Figure S10

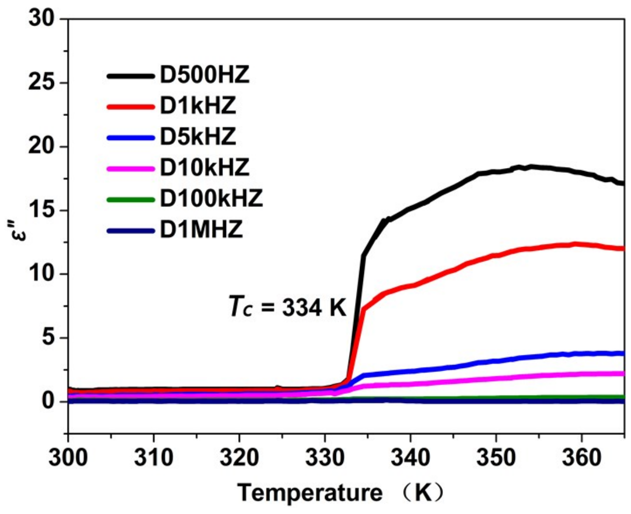

Figure S11

Please do not adjust margins

Please do not adjust margins

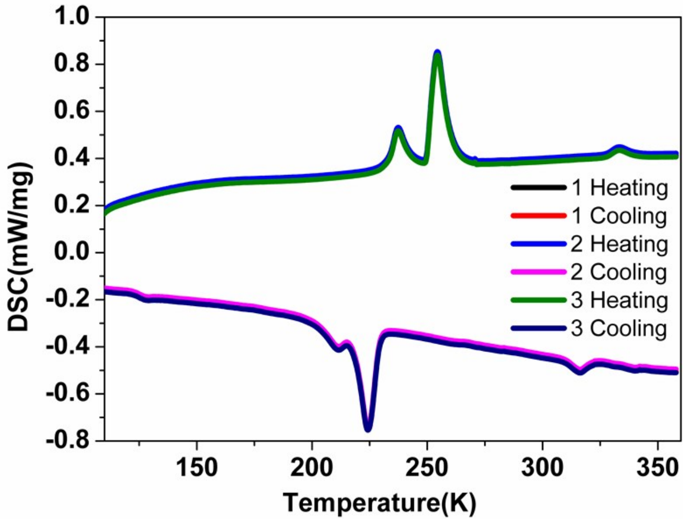

Figure S12

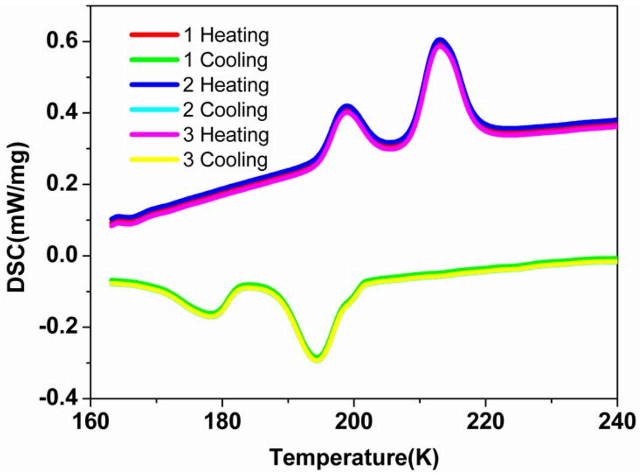

Figure S13

This journal is © The Royal Society of Chemistry 2020

Please do not adjust margins

Please do not adjust margins

Table S1. Summary of crystallographic data for complex **1**

**1**	δ phase (200 K)	β *Phase* (303K)	α phase (348K)
Empirical formula	$C_{10}H_{24}N_2Cl_4Mn$	$C_{10}H_{24}N_2Cl_4Mn$	$C_{10}H_{24}N_2Cl_4Mn$
Formula weight	369.05	369.05	369.06
T, K	200.00(10)	303	348(2)
Crystal system, space group	Monoclinic, $P2_1/c$	Orthorhombic, *Pnma*	Orthorhombic, *Pnma*
a, Å	18.2344(10)	13.1299(9)	13.1016(14)
b, Å	12.6038(4)	8.8855(10)	9.0403(12)
c, Å	18.1345(10)	16.1959(14)	16.1804(19)
β, deg	119.224(7)	90	90
V, Å^{-3}	3637.2(4)	1889.5(3)	1916.4(4)
Z	8	4	4
$D_{calc.}$, mg m^{-3}	1.348	1.297	1.283
μ, mm^{-1}	11.178	1.249	1.232
F (000), e	1528.0	764.0	768.0
2ϑ range, deg	5.554 → 154.514	3.994 → 50.994	4 → 60.202
hkl range	$-22 \leqslant h \leqslant 22$	$-15 \leqslant h \leqslant 15$	$-15 \leqslant h \leqslant 17$
	$-15 \leqslant k \leqslant 15$	$-9 \leqslant k \leqslant 10$	$-11 \leqslant k \leqslant 11$
	$-22 \leqslant l \leqslant 22$	$-19 \leqslant \mathrm{l} \leqslant 18$	$-20 \leqslant l \leqslant 20$
Data/Restraints/parameters	12898/221/320	1823/227/121	2365/166/139
Reflections collected/unique	12898 / 12898	10208 / 1823	10811 / 2365
R_{int}	0.0326	0.0486	0.0472
Goodness-of-fit on F^2	1.089	1.148	1.086
R_1/wR_2 [$I > 2\sigma(I)$]	0.0655 / 0.1787	0.0913 / 0.2426	0.0915 / 0.2407
R_1/wR_2 (all data)	0.0809 / 0.2027	0.1323 / 0.2602	0.1622 / 0.2765
Largest peak and hole (e Å^{-3})	0.58/-0.58	0.83/-0.66	0.55/-0.40

This journal is © The Royal Society of Chemistry 2020

Please do not adjust margins

Please do not adjust margins

Table S2. Selected bond lengths and bond angles of complex **1** at 200 K

Bond lengths	Å	Bong angles	deg
Mn01-Cl03	2.370(18)	Cl03-Mn01-Cl06	111.10(7)
Mn01-Cl06	2.375(2)	Cl09-Mn01-Cl03	109.17(8)
Mn01-Cl09	2.362(18)	Cl09-Mn01-Cl06	108.20(8)
Mn01-Cl0A	2.353(18)	Cl0A-Mn01-Cl03	111.57(8)
Mn1_5-Cl2_5	2.353(19)	Cl0A-Mn01-Cl06	107.55(8)
Mn1_5-Cl3_5	2.361(17)	Cl0A-Mn01-Cl09	109.17(9)
Mn1_5-Cl4_5	2.344(19)	Cl2_5-Mn1_5-Cl3_5	107.54(7)
Mn1_5-Cl1_5	2.358(18)	Cl2_5-Mn1_5-Cl1_5	107.99(8)
C4_2-C5_2	1.299(9)	Cl4_5-Mn1_5-Cl3_5	112.97(9)
C4_3-C5_3	1.294(8)	Cl4_5-Mn1_5-Cl3_5	109.29(7)
C4_1-C5_1	1.304(7)	Cl4_5-Mn1_5-Cl1_5	109.12(8)
C4_4-C5_4	1.281(9)	Cl1_5-Mn1_5-Cl3_5	109.89(8)

Table S3. Selected bond lengths and bond angles of complex **1** at 303 K

Bond lengths	Å	Bong angles	deg
Mn1-Cl1	2.303(10)	Cl2-Mn1-Cl3	107.8(14)
Mn1-Cl1A	2.342(4)	Cl1-Mn1-Cl2	114.1(15)
Mn1-Cl2	2.306(10)	Cl1-Mn1-Cl3	115.0(15)
Mn1-Cl2A	2.335(4)	Cl3A-Mn1-Cl1A	112.4(5)
Mn1-Cl3	2.311(10)	Cl2A-Mn1-Cl1A	107.6(3)
Mn1-Cl3A	2.341(5)	Cl2A-Mn1-Cl3A	111.9(4)
C3-C4	1.193(9)	Cl1$^{\#1}$-Mn1-Cl2	114.1(15)
C7-C8	1.209(9)	Cl1$^{\#1}$-Mn1-Cl3	115.0(15)

Symmetry transformations used to generate equivalent atoms: $^{\#1}$ +*X*, -1/2-*Y*, +*Z*

Table S4. Selected bond lengths and bond angles of complex **1** at 348 K

Bond lengths	Å	Bong angles	deg
Mn1-Cl3	2.325(3)	Cl3-Mn1-Cl2	111.62(16)
Mn1-Cl1	2.332(2)	Cl3-Mn1-Cl1	108.20(11)
Mn1-Cl2	2.353(3)	Cl1-Mn1-Cl2	107.94(10)

Please do not adjust margins

Please do not adjust margins

C4_2-C5_2	1.337(9)	C4_1-C5_1	1.342(9)

Table S5. Summary of crystallographic data for complex **2**

**2**	*γ* phase (173 K)	*α* phase (223 K)
Empirical formula	$C_{10}H_{24}N_2Br_4Mn$	$C_{10}H_{24}N_2Br_4Mn$
Formula weight	546.89	546.89
T, K	173(2)	223(2)
Crystal system, space group	Monoclinic, $P2_1/c$	Orthorhombic, *Pnma*
a, Å	9.2339(6)	13.4500(3)
b, Å	15.1859(10)	9.1134(2)
c, Å	13.7409(8)	16.4176(5)
β, deg	91.494(6)	90
V, $Å^{-3}$	1926.2(2)	2012.40(9)
Z	4	4
$D_{calc.}$, mg m^{-3}	1.886	1.805
μ, mm^{-1}	8.969	14.522
F (000), e	1052	1052.0
2ϑ range, deg	3.998 → 50.018	8.498 →153.886
hkl range	$-10 \leqslant h \leqslant 10$	$-11 \leqslant h \leqslant 16$
	$-18 \leqslant k \leqslant 18$	$-10 \leqslant k \leqslant 11$
	$-16 \leqslant l \leqslant 16$	$-20 \leqslant l \leqslant 20$
Data/Restraints/parameters	3390 / 0 / 160	2135/113/96
Reflections collected/unique	15699 / 3390	7663 / 2135
R_{int}	0.1208	0.0430
Goodness-of-fit on F^2	1.121	1.051
R_1/wR_2 [$I > 2\sigma(I)$]	0.1271/ 0.2087	0.1182 / 0.2712
R_1/wR_2 (all data)	0.1680 / 0.2253	0.1417 / 0.2835
Largest peak and hole (e $Å^{-3}$)	1.50 / -0.72	1.24/-0.80

Please do not adjust margins

Table S6. Selected bond lengths and angles of complex **2** at 173 K

Bond lengths	Å	Bong angles	deg
Br1-Mn1	2.505(3)	Br4-Mn1-Br1	118.26(12)
Br2-Mn1	2.535(3)	Br4-Mn1-Br3	105.57(11)
Br3-Mn1	2.510(3)	Br1-Mn1-Br3	107.14(11)
Br4-Mn1	2.500(3)	Br4-Mn1-Br2	106.37(11)
C4-C5	1.29(3)	Br1-Mn1-Br2	105.50(11)
C9-C10	1.26(3)	Br3-Mn1-Br2	114.36(12)

Table S7. Selected bond lengths and angles of complex **2** at 223 K

Bond lengths	Å	Bong angles	deg
Br1-Mn1	2.472(2)	Br1-Mn1-Br2	107.50(10)
Br3-Mn1	2.474(3)	Br1-Mn1-Br3	108.14(9)
Br2-Mn1	2.502(4)	Br3-Mn1-Br2	112.05(15)
C3-C4	1.221(18)	Br1$^{\#1}$-Mn1-Br2	107.50(10)
C8-C7	1.263(17)	Br1$^{\#1}$-Mn1-Br1	113.60(15)

Symmetry transformations used to generate equivalent atoms: $^{\#1}$ +*X*, 1/2-*Y*, +*Z*
